# Time- & Load-Dependence of Triboelectric Effect

**DOI:** 10.1038/s41598-018-20937-6

**Published:** 2018-02-06

**Authors:** Shuaihang Pan, Nian Yin, Zhinan Zhang

**Affiliations:** 1 School of Mechanical & Aerospace Engineering, University of California, Los Angeles, 90095 USA; 2 School of Mechanical Engineering, Shanghai Jiao Tong University, Shanghai, 200240 China; 3 State Key Laboratory of Solid Lubrication, Lanzhou Institute of Chemical Physics, Chinese Academy of Science, Lanzhou, 73000 China

## Abstract

Time- and load-dependent friction behavior is considered as important for a long time, due to its time-evolution and force-driving characteristics. However, its electronic behavior, mainly considered in triboelectric effect, has almost never been given the full attention and analyses from the above point of view. In this paper, by experimenting with fcc-latticed aluminum and copper friction pairs, the mechanical and electronic behaviors of friction contacts are correlated by time and load analyses, and the behind physical understanding is provided. Most importantly, the difference of “response lag” in force and electricity is discussed, the extreme points of coefficient of friction with the increasing normal loads are observed and explained with the surface properties and dynamical behaviors (i.e. wear), and the micro and macro theories linking tribo-electricity to normal load and wear (i.e. the physical explanation between coupled electrical and mechanical phenomena) are successfully developed and tested.

## Introduction

Friction, although discovered and understood since the ancient times, is still a mysterious field, awaiting more integrated exploration and complete studies. With the development of more delicate equipment and more accurate experiments, the strongly coupled characteristics of friction is now being analyzed, which tries to answer the essential questions in tribology from diversified viewing points, e.g. mechanics, electronics^1^, photonics^2^, and thermodynamics^3^.

Due to the ubiquitous nature of triboelectricity, triboelectronics, mainly concerned with triboelectric effect, is one of the most heatedly researched fields, especially after triboelectric nanogenerator (TENG)^1,4^ has been invented, and more efforts are put into this area for solid explanations^5,6^. With the growing complexity of the triboelectric systems in use, because of the highly entangled material behavior inside, the fundamental understanding of triboelectric effect is still open to question. However, it is a huge pity that some people believe that the research into triboelectric effect on elementary friction pairs is already sufficient.

Very recently, the studies on tribology goes to micro or nanoscale, which yields a lot of new, or even surprising, findings (e.g. friction is now considered to be contact area-dependent^7^). Step by step, it’s the time-dependence and load-dependence in friction contact that refreshes the traditional notions about tribo-behaviors^8^. However, the shallow understanding of triboelectric effect seems to ignore this new trend in tribological analyses, and therefore doesn’t include any discussion of possible time- and load-dependence of electronic behavior in friction contact. No doubt that this research lag causes much confusion in revealing the true essence of triboelectric effect.

This paper goes back to the fundamental study in friction to link mechanical and electronic behavior inside. By using fcc-structured aluminum and copper to form the friction pairs (thanks to their great availability, high machinability, and potential applications in tribo-devices^9,10^), we compare the difference in mechanical (i.e. coefficient of friction, CoF) and electronic responses (i.e. triboelectric current), which guides us to observe the response lag in different time phase of friction contact (which indicates the time-dependence); then, by looking into the trend in CoF and steady-state fluctuations, we confirm the load effect on friction process and illustrate the competitive mechanisms in governing the change in CoF; finally, tribo-current is systematically studied, by which the experiment proves the load-dependence of triboelectric effect, and the results are compared with the existent theories for clarification in details.

In general, this paper links the mechanical and electronic phenomena in friction, and discusses the time- & load-dependence of the friction process for the first time. Since the study is fundamental by all means, given the simple-latticed materials, the results are suitable be to the basis for more fundamental research in tribology and triboelectricity.

## Results and Discussion

### Time-dependence

**Figure 1:**
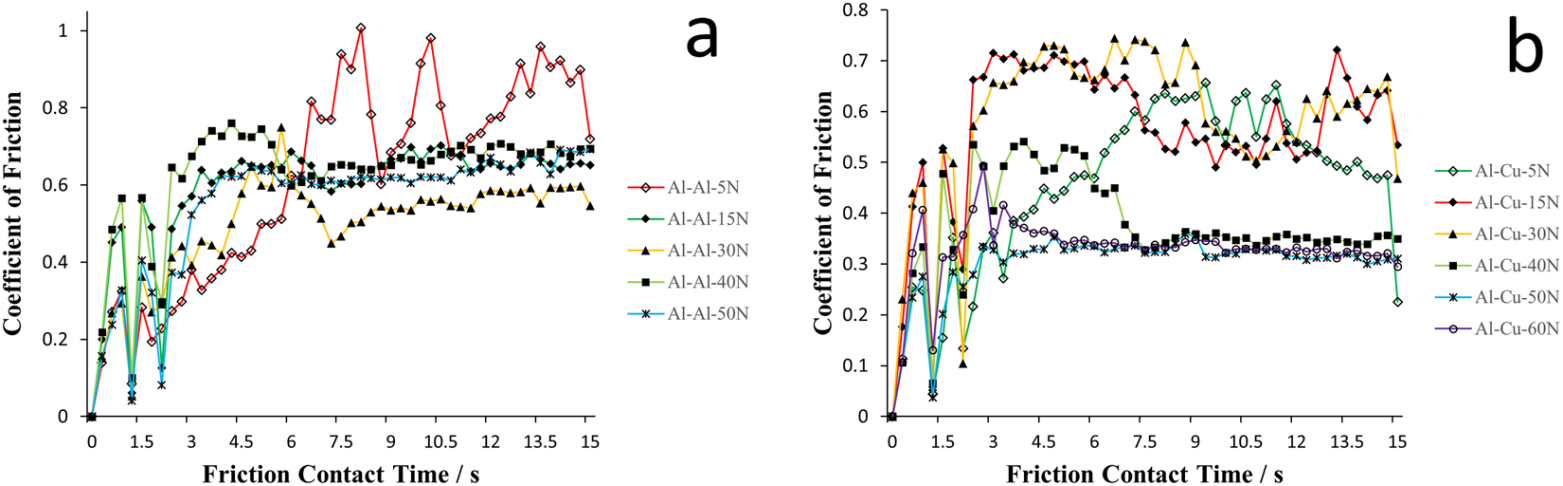
CoF in relation to friction contact time for (**a**) Al-Al friction pair and (**b**) Al-Cu friction pair under 5 N, 15 N, 30 N, 40 N, 50 N, and 60 N.

**Figure 2:**
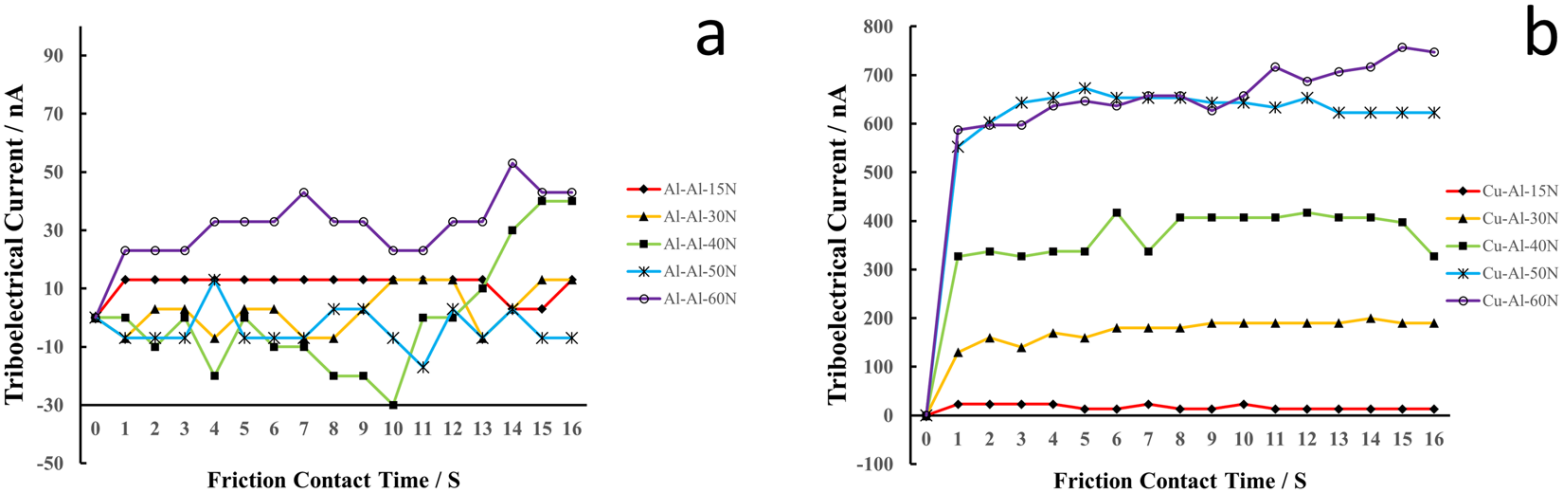
The triboelectric current during friction contact for (**a**) Al-Al and (**b**) Al-Cu friction pairs under 15 N, 30 N, 40 N, 50 N, and 60 N.

The experiment results are shown in Figs 1 and 2. Figure 1 shows the time-dependent triboelectric current of Al-Al and Al-Cu friction pair under 15 N, 30 N, 40 N, 50 N, and 60 N. The reason for setting the 5 N data all as 0 is due to the negligible triboelectric effect under such a normal load, and the triboelectric current is only comparable to the noise.

Coefficient of friction is an important indicator of the friction behavior of the interfaces, since it can demonstrate the force responses and friction severity. Moreover, we should note that the time range for measurement of triboelectric current is reaching to more than 15 seconds in the experiments. This is because once the friction contact is stopped, the mechanical signal (namely the CoF data) will immediately stop accordingly, but the electrical signal (namely the triboelectric current) won’t be so, due to the built-up voltage vanishing delay.

Based on the CoF results shown in Fig. 1, we can see that after about 4.5 s, the friction force response will be stabilized, whereas the electricity will stabilize immediately (<1 s) after the friction contact starts, as demonstrated in Fig. 2. Why the mechanical and electrical signals are not synchronized is due to the trigger mode difference. For triboelectricity, once 2 surfaces contact and deform, charge will start to accumulate, voltage builds up;^11^ however, if there exists no relative movement, friction force won’t be introduced. In this sense, electrical signal is a leading feedback, whereas force is a synchronous signal to friction contact.

For the same reason, why the triboelectric current can last even after the relative motion can also be answered.

This non-synchronization between friction force and electrical current in response to friction contact should be treated importantly. In friction monitoring, it is then better to use electrical signal as the indicator, because the lag in the force response in friction contact will cause the error and deviation from proper control. Indeed, this is why many friction studies yield different trends and understandings in even the simplest cases of friction pairs.

### Load dependence of Coefficient of Friction

**Figure 3:**
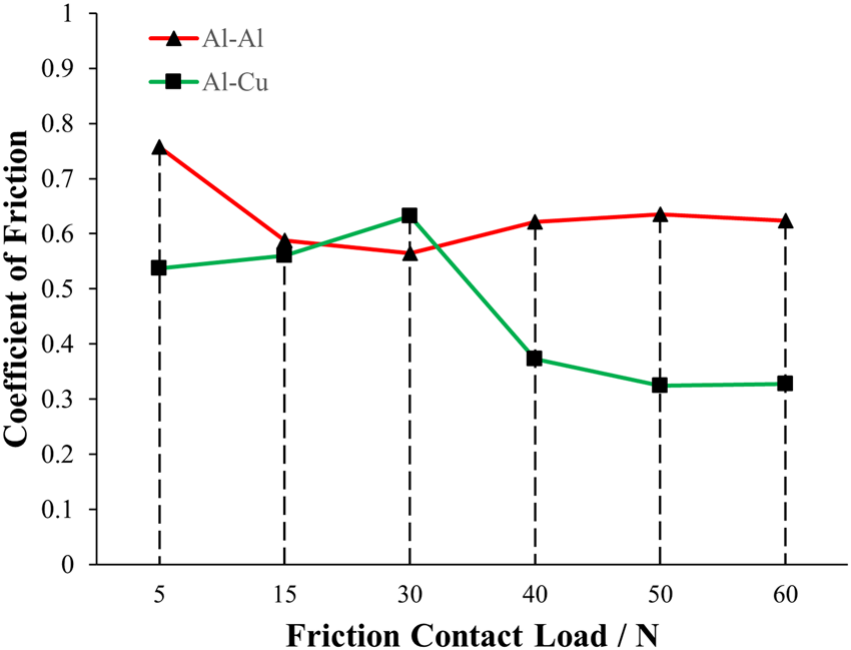
The averaged stabilized CoF of Al-Al and Al-Cu pairs under different applied normal loads.

Via the time-dependent CoF illustrated by Fig. 1, the results of the averaged stabilized CoF are depicted in Fig. 3. By “averaged stabilized”, we analyze the data from ~4.5 s.

First, we should consider the real-time variation of CoF of each case in Fig. 1 and the statistical results of CoF in Fig. 3. Obviously, no matter in Al-Al or Al-Cu friction pairs, with the increase of the normal loads from 5 N to 60 N, the larger normal load will yield a more stable CoF response. This result is further made evident by calculating the standard deviation of stabilized CoFs, as shown in Table 1. So, in order to get the uniform and low-noise performance in friction process, a relative large normal load is expected. Physically speaking, the trend of standard deviation in CoFs is natural by considering the surface roughness. With the increase of the loads, surface is subject to more deformation and noise from surface roughness will be suppressed by friction as a medium for surface tuning and wear as a way of polishing^12,13^. This has never been discussed in detail in previous studies, and the schematic illustration is shown in Fig. 5.

**Table 1:** The standard deviation of stabilized CoF under different applied normal loads.

Load/N	Standard Deviation of COF	
	Al-Al Friction Pair	Al-Cu Friction Pair
5	0.219	0.112
15	0.088	0.074
30	0.077	0.074
40	0.068	0.073
50	0.053	0.015
60	0.054	0.020

Besides, when we revisit Figs 1 and 3, another important trend is noticed that there is always an extreme point with the increase of normal loads from 5 N to 60 N (i.e. from 0.166 MPa to 1.987 MPa). CoFs are load-dependent. This is a long-standing and widely accepted phenomenon^14–16^. However, the extreme points illustrate the competing mechanisms in CoF evolution with different normal loads^14–16^. The change of COF is a competing balance among surface roughness, materials’ properties (hardness & young’s modulus), and dynamical friction behavior (whether wear dominates).

**Figure 4:**
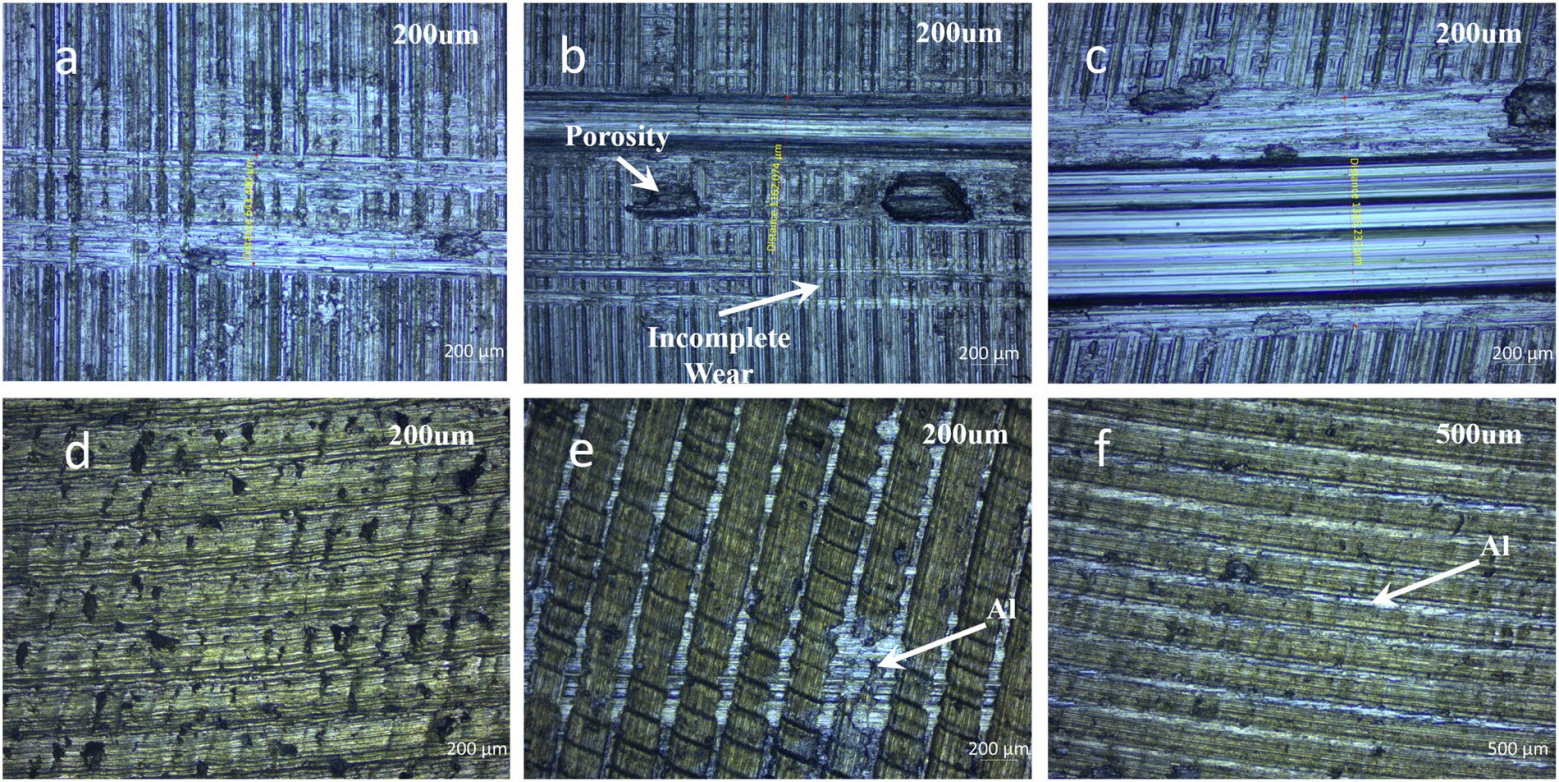
The microscopy for the surface topography of the friction pairs of (**a**) Al-Al under 5 N; (**b**) Al-Al under 30 N; (**c**) Al-Al under 50 N; (**d**) Al-Cu under 5 N; (**e**) Al-Cu under 30 N; (**f**) Al-Cu under 50 N. (The lighter, brighter phase is Al, whereas the darker, more yellowish phase is Cu). (Note: All pictures are taken along the reciprocating motion direction of the friction pairs).

**Figure 5:**
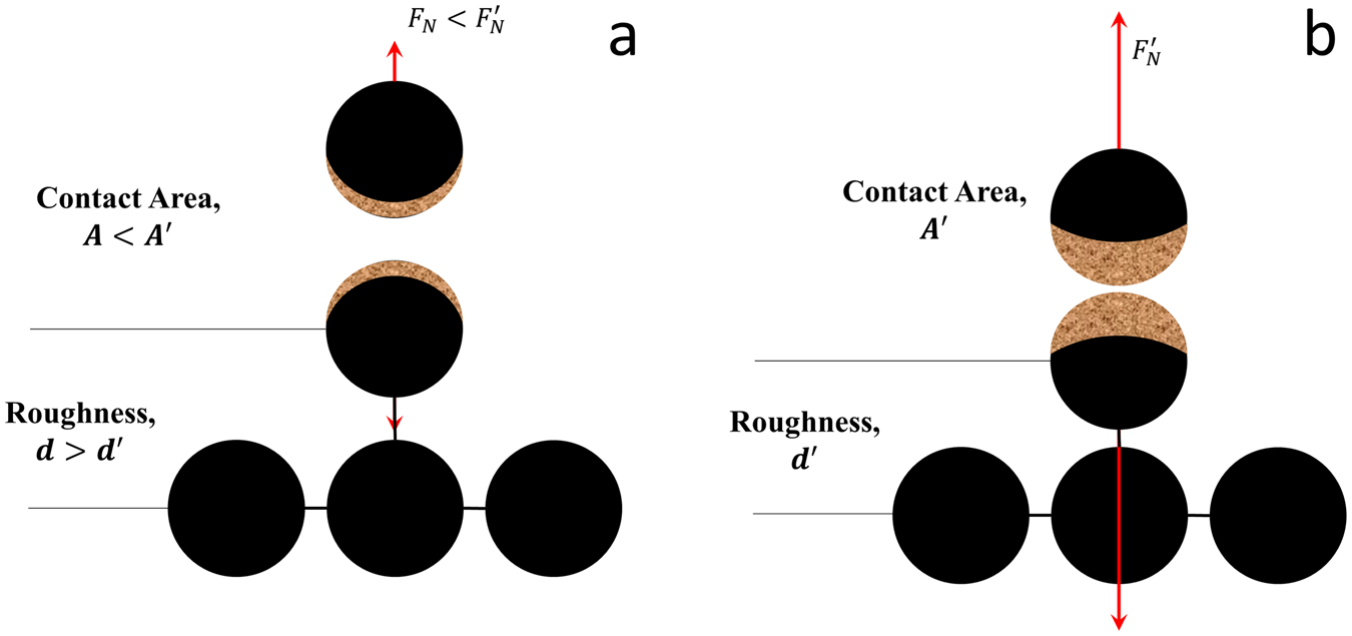
The descriptive illustration of surface configuration under different normal loads **(a)**. When the normal load is small, the roughness is hardly removed, and the effective contact area is small; **(b)** When the normal load is large, the roughness will be suppressed (or even removed), and the effective contact area is large. (*F*_*N*_ indicates the interactive force between the 2 surfaces).

The surface topographies after the Al-Al friction contact under 5 N, 30 N, and 50 N are shown in Fig. 4a–c: The contact region widths are increased from 640 um to 1160 um and finally to 1315 um. For Al-Al friction pairs, there is no difference in hardness and young’s modulus in the 2 surfaces, so when the larger load is exerted, surface roughness will first be suppressed due to the similar hardness and young’s modulus of the two surfaces, which gives a slightly smaller CoF at the beginning^14^ (this reasoning can be proven by the reduction of stabilized CoF fluctuation in Fig. 1; besides, under medium normal loads, we can still see larger porosities after roughness removal to reduce the contact area, and the incomplete wear actually changes the sliding friction into quasi-rolling friction, since the interlaced trace is left, as indicated by Fig. 4b); When the load is still larger, wear will dominate. (This isn’t discussed in ref.^14^., etc.) In this sense, adhesive force between 2 layers will dominate, as demonstrated by Fig. 4; Besides, since wear is prevalent on the interface, the increasing area of friction contact will also add to the increase of CoF (See Fig. 5)^16,17^. Therefore, when the normal load is larger (i.e. 50–60 N in our experiment), CoF will be greater slightly, and CoF minimum will exist for Al-Al friction pair.

On the other hand, for Al-Cu friction pair, whose results are shown in Fig. 4d–f, hardness and young’s modulus are quite different (for Al, Mohr hardness is 2.75 and young’s modulus is 70 GPa; for Cu, Mohr hardness is 3.00 and young’s modulus is 110–128 GPa). This indicates that even after the severe wear of Al onto Cu, Cu surface roughness won’t be changed too much. This understanding can be proven mainly by Fig. 4d, and the clear attachment of Al onto Cu (whose surface roughness in the form of the grinding textures isn’t changed too much) in Fig. 4e and f can also show the role of the different hardness. Under this circumstance, when normal load is increased, increased adhesive force and wear-introduced contact area will first play a role, which leads to the result of a slightly bigger CoF. (See Fig. 4d–e.) If normal load continues to increase (i.e. from 30–40 N to 50–60 N), the roughness of Cu surface will finally be smoothened (confirmed by the blurring of the Cu roughness structure on surface in Fig. 4f), and a relatively small CoF will be observed. The above reasons result in the maximum CoF point in Al-Cu friction pair.

In brief, for homogeneous friction pair like Al-Al interface, with the increase of the normal load, CoF will show a minimum point, since smoothened roughness comes first due to similar surface properties, followed by dominant wear behavior and contact area increase; for heterogeneous friction pair like Al-Cu interface, the CoF maximum point is to be observed with the increase of the normal load, and the dominance of wear behavior first and then smoothened roughness in sequence is the fundamental reason. This mechanical behavior and observation is of great importance, because it can introduce “the point effect” which changes the triboelectric performance by tuning the CoF and contact area^18,19^.

At the same time, we have to admit that there is limitation for our findings: Our success in observing the CoF trend difference is based on the similarity of Al and Cu lattices (both are fcc lattice), and it may be more complex if the lattice structure plays a role in friction process (especially when commensurate plane is included for discussion^20,21^); In addition, no chemical reaction happens between heterogeneous interfaces, which indicates that the extreme-point trend may not hold for chemically reactive interface like Al-Ti, Cu-Ag interfaces.

### Load dependence of Triboelectricity

**Figure 6:**
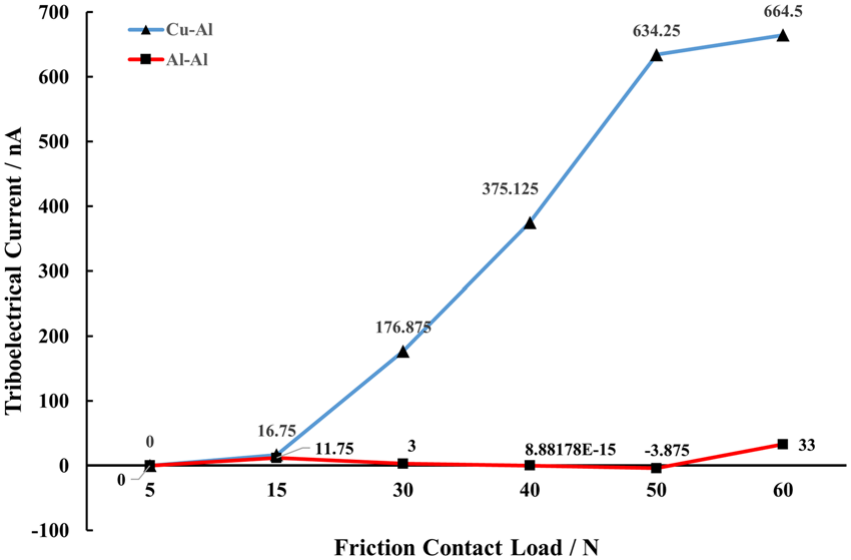
The averaged triboelectric current of Al-Al and Al-Cu pairs in relation to friction contact loads.

Due to the ubiquitous triboelectricity, only understanding friction behavior of two surfaces on its mechanical aspect is insufficient. The load-dependent trend in tribo-current is depicted in Fig. 6.

First, the tribo-series of Al and Al must be the same, so it is natural that there exists no obvious triboelectricity. The positive and negative small-to-nothing current of Al-Al friction pair proves the randomness of the detected triboelectricity, which indicates that there will be no net triboelectric effect.

Then, we have to look at the Al-Cu friction pair more closely. As clarified by many marvelous recent studies, triboelectricity can be triggered by both electron transport and ion/material transport. For metal-metal contact case (in which regular lattice can be expected), electron transport will play an important role. With the increase of the normal load, lattice deformation will be introduced more^22,23^. Thus, the phonon energy modes will be extended to trigger electrons to transfer in a more effective way, resulting in a more significant steady triboelectric current^5,6^, by the functional linear relationship of:

1 $${{I}}_{{electron}}\propto {{D}}_{{s}}\propto {f}({{F}}_{{N}})$$

Where *D*_s_ denotes the surface density of states; in our case, it’s only a function of *F*_*N*_, since only normal loads are changed.

Of course, pure metal-metal contact region will form, and the Fermi level will exert certain effect both thermally and electronically^24–26^.

The non-linearity introduced by 15–50 N normal loads in Al-Cu pairs can be understood by the following discussion: Due to the wear behavior at the interface and the roughness removal, there is constant ion/material transport accompanying the pure electron transport, which adds to the non-linearity of tribo-electricity with respect to normal load. Wear is indeed an efficient bond-breaking and bond-forming process in between the 2 involved surfaces^27^. For metal cases, the (nearly) free electrons and metal cores can be transferred via different routes with different amounts, due to the isotropic ionic bonds in between atom cores and electrons. So, the ions can be Al^3+^ or/and Cu^2+^, etc. once their electrons have been peeled off to transfer and then they are worn between the interface^27–29^.

More interestingly, the approximate exponential curve of the tribo-current corresponds to the wear removal rate proposed by Jacobs, *et al*. in ref.^30^. This demonstrates the correspondence between tribo-current and wear rate (both of which are time-derivative), as shown in equation (2).

2 $${{I}}_{{ion}}\propto {\dot{{N}}}_{{ion}}\propto {\dot{{N}}}_{{wear}}$$

Since ${\dot{N}}_{wear}=\dot{N}({\rm{S}}1\leftrightarrow {\rm{S}}2)\cdot \exp (\frac{{P}_{compression}\cdot {\rm{\Delta }}V}{{k}_{B}T})$, this means that ${I}_{ion}=I\cdot \exp (\frac{{P}_{compression}\cdot {\rm{\Delta }}V}{{k}_{B}T})$ should be expected^30^.

Here, ${\dot{N}}_{ion}$ is the ion transfer rate between the 2 surfaces, whereas ${\dot{N}}_{wear}$ the wear rate. ${\dot{N}}_{wear}$ isn’t totally equal to, but is only proportional to ${\dot{N}}_{ion}$, because some atom transferring may not carry charges with it. $\dot{N}$ is the intrinsic characteristic wear rate of a specific friction pair, which is a function of properties of friction surface S1 and surface S2.

*P*_*compression*_ demonstrates the role played by friction contact normal load, which can be calculated by.

3 $${{P}}_{{compression}}=\frac{{{F}}_{{N}}}{{{A}}_{{real}}}\cong \frac{{{F}}_{{N}}}{{A}}$$

Where *F*_*N*_ is the normal load. *A*_*real*_ refers to the actual contact area, but in real application, without loss of generality, *A*, as the visual contact area, can be used.

${\rm{\Delta }}V$ should be the characteristic volume in wear process. In this case, the material is removed and transferred on the basis of lattice level, since no chemical reactions are significant. Hence, ${\rm{\Delta }}V$ can be calculated by equation (4).

4 $${\rm{\Delta }}V={\bar{{a}}}^{3}$$

Where $\bar{a}$ should be the mean lattice parameter (or equivalent lattice parameter) for the friction pair. For Al (4.046 Å)-Cu (3.597 Å) friction pair, it can be calculated with 3.8 Å.

One thing we should note is that wear is also an observable macro process to remove and smoothen the roughness on the surface. In this sense, ${\rm{\Delta }}V$ may also be used to show the removal heterogeneity on normal direction with the above physical meaning, i.e. ${\rm{\Delta }}V$ can be expressed as.

5 $${\rm{\Delta }}V={\bar{{a}}}^{2}\cdot \frac{{{R}}_{{a}}}{2}$$

**Figure 7:**
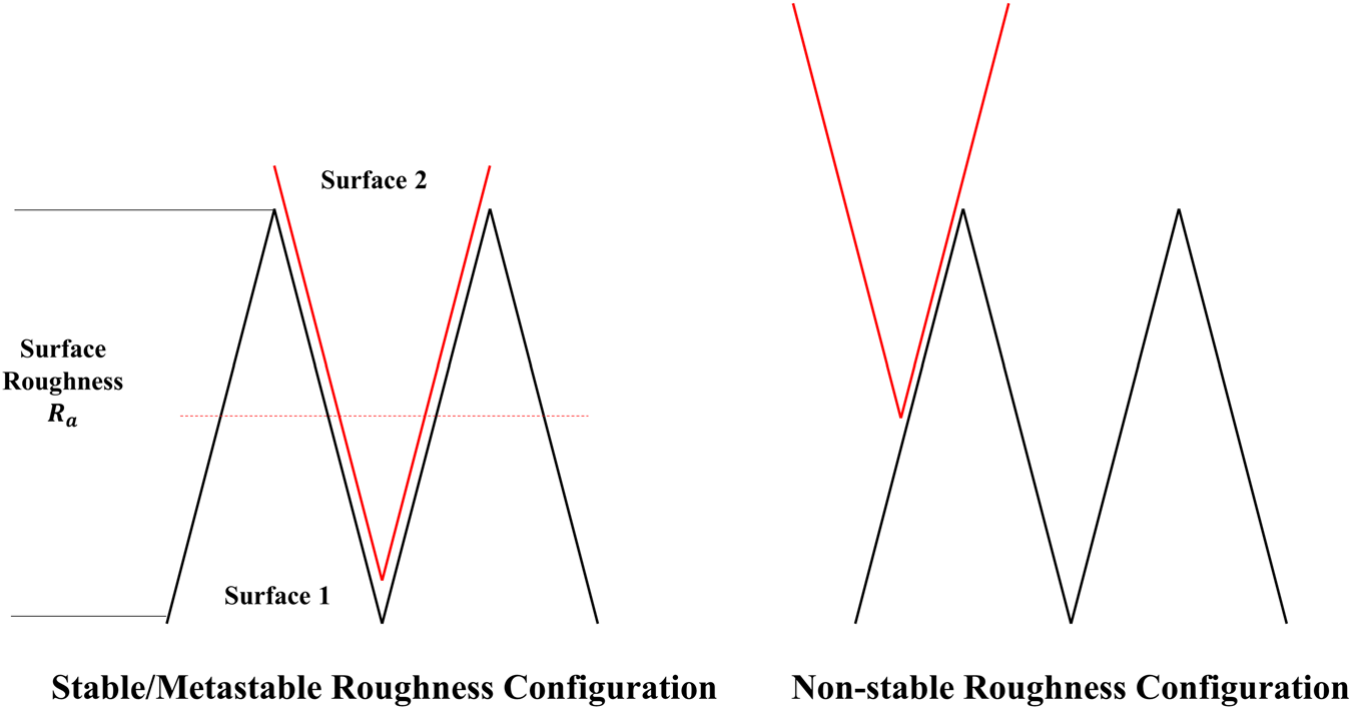
The illustration of roughness configuration under thermo-stability consideration (“Stable” indicates “minimization of surface”).

Where *R*_*a*_ is the surface roughness (i.e. 1.6 um in our experiment). Why $\frac{{R}_{a}}{2}$ is more accurate is because the roughness need to be shared by 2 surfaces, if the structure is to be stable in configuration, as indicated in Fig. 7. This is based on the thermodynamics consideration. Surface usually has higher energy (especially for metals with astounding surface energies), and the system will try to minimize its surface to transfer into a more stable state^27,29^. Apparently, the surface roughness and the asperities will be more stable, if they match with each other, due to the reduced exposed surface area, as vividly shown in Fig. 7.

By applying the exponential law to simulate the curve obtained in Fig. 6, we can get the leading factor of I as 4.78 nA. If we consider the *micro-explanation* from lattice point, then, with the help of the lattice parameter, in a physical sense, the calculated tribo-current should be.

6 $${{I}}_{{ion}}=4.78\cdot {{e}}^{4.39\times {10}^{-4}\cdot {{F}}_{{\boldsymbol{N}}}}({nA})$$

If we look at the tribo-current from the *macro-explanation*, given the surface configuration, from macro point of view, the calculated tribo-current should be.

7 $${{I}}_{{ion}}=4.78\cdot {{e}}^{0.924\cdot {{F}}_{{\boldsymbol{N}}}}({nA})$$

**Figure 8:**
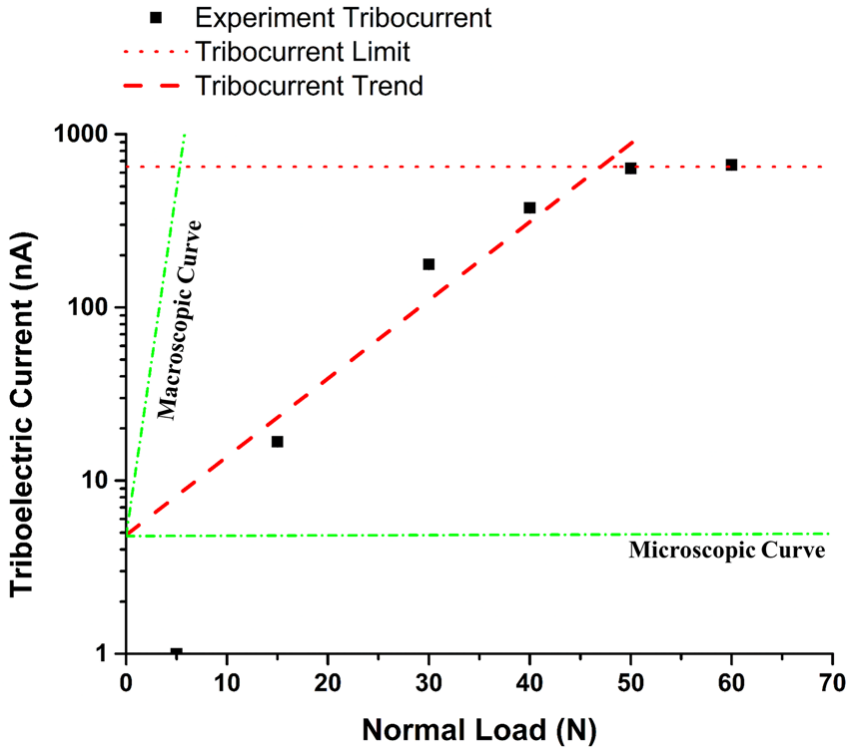
The comparison between tribo-current data from experiment with Al-Cu friction pair, microscopic analysis and macroscopic analysis.

The results from equations (6) and (7), and Fig. 6 is depicted in Fig. 8. Our experiment data reside right in between the region enclosed by micro (predicted from lattice) and macroscopic (predicted from roughness) tribo-current, which proves the rightness of our experiment and discussion. Generally speaking, ion transfer to introduce triboelectric effect is a combination of micro and macro phenomena. The mismatch possibly comes from the following effects: 1) Roughness is dynamically changed, as shown in Fig. 4, which adds to the nuance between the theoretical and experimental data; 2) Contact area is also variable, which provides different size of channel for charge transfer^18,19^.

Besides, we should mention that it’s a robust analytical process, if we use macro theory to predict the triboelectric behavior. The main reason is that surface roughness will be suppressed to different extent under different loads (i.e. ${R}_{a}\downarrow$), wear will serve as a polishing process (i.e. ${R}_{a}\downarrow$), and the increasing contact area will reduce the actual compression pressure (i.e.$\,{A}_{real}\uparrow$ and ${P}_{compression}\downarrow$)^28,31^, as clarified in the discussion for CoF and equation (3). This understanding can better help limit the deviation of triboloelectric experiment from pre-experiment calculation.

Due to the simple regular lattice in our friction system, since the trend is more approaching to lattice-induced ion-transferring tribo-current, we still believe the micro transport phenomena are more dominant in triboelectric effect. Under this assumption, only by combining the current by electron transport and the current by ion transport can we match the tribo-current with our experiment data more precisely.

Last but not the least, when the normal load is extremely large (i.e. 50–60 N in our case), the tribo-current will be impeded and saturated, as shown in Fig. 8. This is because the electric charge built on the interface will experience the dynamical breakdown, when the friction contact brings the surface contact at a very small distance^3,6^.

## Conclusion

In this paper, the Al-Al and Al-Cu friction pairs’ tribological behavior is analyzed under different loads within a certain time range. The difference between friction force and triboelectric current is the response lag before or after the friction contact, since the contact electrification will play a role in interfacial current formation.

Then, by analyzing the force responses and its trend in relation with load, the competition mechanisms (i.e. surface smoothening and contact area increase) governing the change of CoF is analyzed: The CoF decreasing effect by surface smoothening and the CoF increasing effect by contact area increase are interacting to vary CoF under different normal loads for a specific friction pair; these effect will give rise to a CoF extreme point, along with the increase of the normal load; whether it’s a maximum (for Al-Cu case) and minimum (for Al-Al case) is dependent on the surface characteristics and properties including material structure, hardness, young’s modulus, etc.

Finally, by considering triboelectric currents in Al-Cu friction pairs (with Al-Al friction pairs as the comparison group), we systematically analyzed the exponential current trend with the increase of normal loads: By the application of the Arrhenius Relationship in wear rate^30^, we give the current range, with the help of micro view into lattice parameter and macro view into surface roughness, which makes this phenomena more predictable; after guaranteeing the physical meaning in our triboelectric trend curve, we point out the micro importance in analyzing triboelectricity, and compensate the deviation from the calculated curve, by incorporating the discussion of electron transport theory.

In summary, the paper is trying to find time- and load-dependence in friction behavior of triboelectric effect. The choice of the fundamental friction pairs, though simple, demonstrates the important relationships between friction force and triboelectricity, with the interaction of materials’ properties, wear rate, and electron/ion transferring process. Of course, more efforts can be invested in future research to investigate the triboelectric performance under the same load with different CoF and contact area, in order for an observation with little mismatch.

## Methods

### Experiment Setup

**Figure 9:**
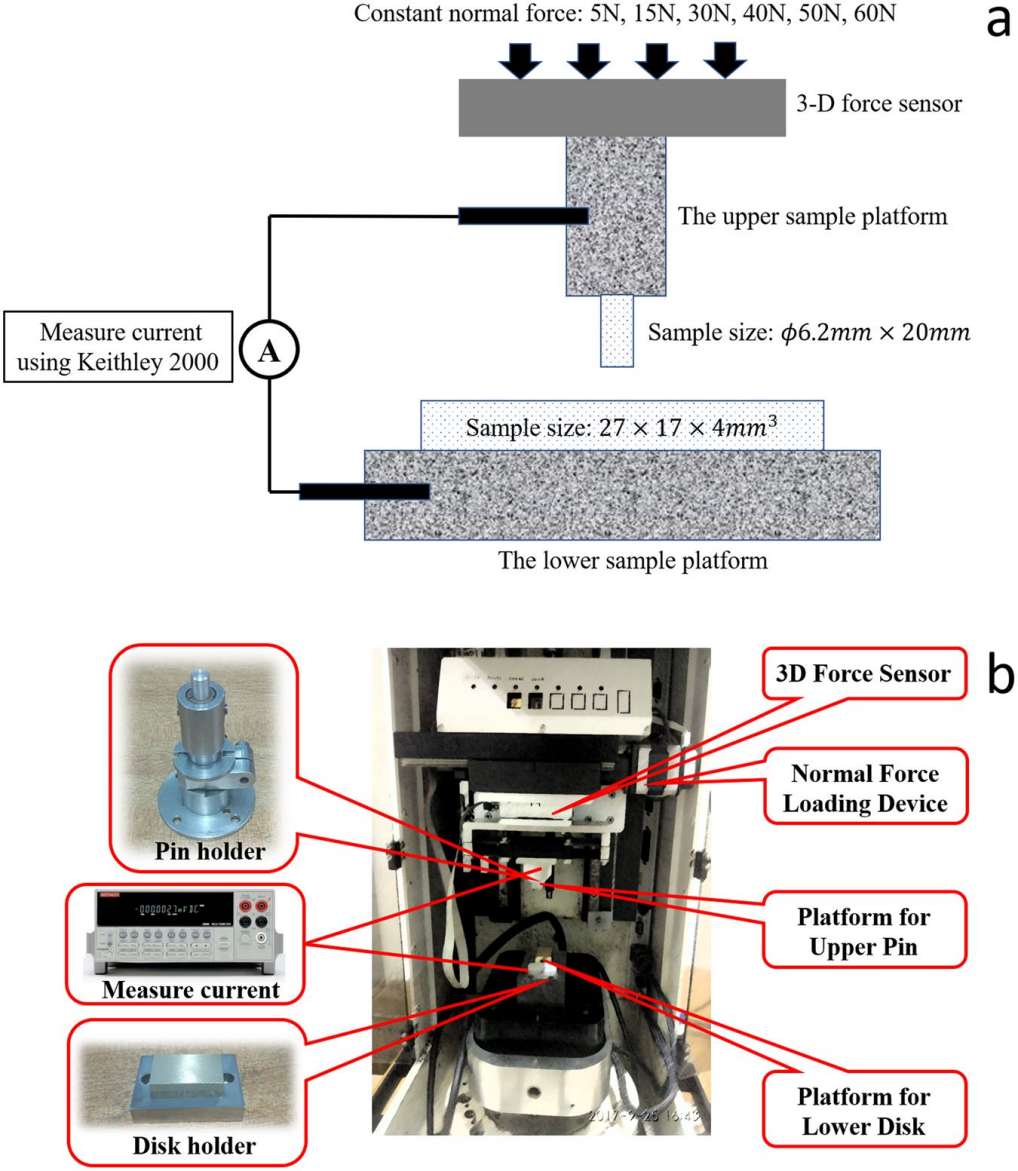
The experiment setup (**a**) The schematic illustration (**b**) UMT-3 Tribometer and the samples (upper pins and lower disks) clamping positions.

We carried out *pin-on-disk friction experiments* on *UMT-3 Tribometer*, the experimental configuration of which is shown in Fig. 9. The pins will be clamped into the upper platform, and the disks are fixed in the lower platform. During the experiments, 5 N, 30 N and 50 N normal forces are exerted onto Al-Al and Al-Cu friction pairs to introduce different friction force. The normal load is applied by the *Normal Force Loading Device*. The pins are moved against the disk surface at the frequency of 2 Hz with the amplitude of 20 mm for 15 s, which is automatically controlled by the computer. The whole experiment is carried out in air at a temperature of ~25 °C.

### Data Collection & Record

At the same time of the experiment, the friction coefficient is measured and recorded by the *3D Force Sensor*; the triboelectric current is recorded during the experiments with *Keithley-2000 Multimeter* (with the resolution of ~10 nA), and the noise of the signal is removed by bench-marking the non-contact current.

### Sample Characterization

After the experiments, the contact surface of the Al and Cu disks are characterized with *photo-microscopy* by *ZEISS Primo Star HD Digital Microscope* (with the advanced distance calibration & measurement) to check the surface topography and surface element composition. The surface element composition is checked to include the reasonable consideration for the effect of surface wear during the friction contacts.

### Sample Preparation

In our experiment, since aluminum and copper are both simple face-center cubic structure (fcc-lattice), we used aluminum (pure, no surface passivation) and copper (pure) to form the friction pairs, in order to reduce the effect from the lattice structure. All the pins are made from aluminum, with diameter of 6.2 mm; the disks are made from copper or aluminum separately, but all are 27 mm in length and 17 mm in width. The pins and disks have the uniform surface roughness of ~1.6 *u*m (provided by the manufacturer), which indicates electron tunneling won’t be a main factor to influence triboelectric current^9^.
